# Epidemiological Analysis of Salmonella enterica subsp. *enterica* Serovar Dublin in German Cattle Herds Using Whole-Genome Sequencing

**DOI:** 10.1128/Spectrum.00332-21

**Published:** 2021-09-15

**Authors:** Silvia García-Soto, Herbert Tomaso, Jörg Linde, Ulrich Methner

**Affiliations:** a Institute of Bacterial Infections and Zoonoses, Friedrich Loeffler Institute, Jena, Germany; Institute of Biomedical Sciences, Universidade de São Paulo

**Keywords:** whole-genome sequencing, *Salmonella enterica* subsp. *enterica* serovar Dublin, bioinformatics, epidemiology, cattle, Germany

## Abstract

Salmonella enterica subsp. *enterica* serovar Dublin is a cattle-adapted serovar that causes enteritis and systemic diseases in animals. In Germany, *S*. Dublin is not detected or is very rarely detected in some federal states but is endemic in certain regions. Information on detailed genetic characteristics of *S.* Dublin is not available. An understanding of the paths and spreading of *S.* Dublin within and between regions and over time is essential to establish effective control strategies. Whole-genome sequencing (WGS) and bioinformatic analysis were used to explore the genetic traits of *S.* Dublin and to determine their epidemiological context. Seventy-four *S*. Dublin strains collected in 2005 to 2018 from 10 federal states were studied. The phylogeny was analyzed using core-genome single-nucleotide polymorphisms (cgSNPs) and core-genome multilocus sequence typing. Genomic clusters at 100 cgSNPs, 40 cgSNPs, and 15 cgSNPs were selected for molecular epidemiology. WGS-based genoserotyping confirmed serotyping. Important specific virulence determinants were detected in all strains, but multidrug resistance in German *S.* Dublin organisms is uncommon. Use of different thresholds for cgSNP analysis enabled a broad view and also a detailed view of the occurrence of *S.* Dublin in Germany. Genomic clusters could be allocated nationwide, to a limited number of federal states, or to special regions only. Results indicate both persistence and spread of *S.* Dublin within and between federal states in short and longer time periods. However, to detect possible routes of infection or persistence of *S.* Dublin indicated by genomic analysis, information on the management of the cattle farms and contacts with corresponding farms are essential.

**IMPORTANCE** Salmonella enterica subsp. *enterica* serovar Dublin is a bovine host-adapted serovar that causes up to 50% of all registered outbreaks of salmonellosis in cattle in Germany. *S.* Dublin is not detected or is only rarely detected in some federal states but has been endemic in certain regions of the country for a long time. Information on genetic traits of the causative strains is essential to determine routes of infection. WGS and bioinformatic analysis should be used to explore the genetic characteristics of *S.* Dublin. Combining the genomic features of *S.* Dublin strains with information on the management of the cattle farms concerned should enable the detection of possible routes of infection or persistence of *S.* Dublin. This approach is regarded as a prerequisite to developing effective intervention strategies.

## INTRODUCTION

Salmonella enterica subsp. *enterica* serovar Dublin is defined as bovine adapted but not bovine restricted and affects calves, young cattle, and adult cattle, causing enteritis and/or systemic disease (1). A wide range of clinical manifestations, from acute clinical disease with high mortality rates and abortions over typhoid-like illness with diarrhea to a chronic carrier state without any clinical signs of infection, may occur (1). Natural infections with the bovine host-adapted serovar *S.* Dublin may occur also in other animal species, particularly small ruminants like sheep and goats (2). Although it is very rare, *S.* Dublin may also cause human infections; because of its high virulence, it is associated with death and thus is considered a global public health concern (3).

In Germany, outbreaks of salmonellosis in cattle herds officially confirmed by a competent authority are notifiable (4). Between 2010 and 2019, approximately 110 to 130 outbreaks of bovine salmonellosis from different serovars were recorded each year. Salmonella enterica subsp. *enterica* serovar Typhimurium was the dominant serovar during this period and caused approximately 40% to 50% of the annually reported cases. *S.* Dublin amounted to 30% to 40% of all registered outbreaks each year. Salmonella enterica subsp. enterica serovar Enteritidis caused approximately 5% to 10% of all outbreaks. All other serovars are summarized and as a group were the cause of 15% to 20% of all outbreaks of German cattle salmonellosis per year. However, there is no single dominant serovar in this group, and no serovar shows an increasing detection rate over longer periods. Compared with *S.* Typhimurium, the cattle-adapted serovar *S.* Dublin is not detected or is rarely detected in some federal states but is endemic in certain regions (5, 6). In Germany, no detailed information is available on the genetic traits of *S.* Dublin isolates from different regions or over longer periods. However, a better understanding of the paths of infection and spreading of the organism within and between regions and over time might give valuable information to establish effective measures to control the disease. Pulsed-field gel electrophoresis (PFGE) has been shown to provide valuable data to separate Salmonella organisms of different serovars (7–9). Whereas a Danish study showed some ability to differentiate between strains of *S.* Dublin (10), results from Sweden have provided unsatisfactory resolution among isolates of this serovar (11). Whole-genome sequencing (WGS) has been used successfully for the characterization of Salmonella organisms belonging to this and other serovars (11–14). Core-genome multilocus sequence typing (cgMLST)- and core-genome single-nucleotide polymorphism (cgSNP)-based typing are widely used for Salmonella genotyping. While cgMLST uses allele numbers for each gene for typing, single-nucleotide polymorphisms (SNPs) are called and used for typing and for building phylogenetic trees in the second approach. Both methods provide a high capability to discriminate between closely related and nonrelated isolates (15). In addition, WGS data give information regarding the genetic context of the isolates, such as virulence genes and antimicrobial resistance (AMR) determinants.

In view of the lack of information regarding the epidemiology of the host-adapted serovar *S.* Dublin in the German cattle population, this study aimed to characterize *S*. Dublin strains that originated from different federal states in Germany in the period between 2005 and 2018. *S.* Dublin organisms isolated during that period were examined phenotypically at the German National Reference Laboratory (NRL) for Salmonellosis in Cattle. WGS and bioinformatic analysis were used (i) to describe the genetic traits of *S*. Dublin strains, (ii) to analyze the phylogeny using cgSNPs as well as cgMLST, and (iii) to compare the results with recent studies on *S*. Dublin genomes in other European countries.

## RESULTS

### Serotyping and antimicrobial susceptibility testing of *S.* Dublin strains.

The isolates were typed according to the Kauffmann-White scheme and revealed the complete antigenic formula (1,9,12:g,p:−) for *S*. Dublin. All 74 *S.* Dublin strains, apart from strain 197, were not resistant to the antimicrobial substances tested (see Data Set S1 in the supplemental material). The MIC values of the *S.* Dublin organisms were as follows: sulfamethoxazole, <512 μg/ml; trimethoprim, <0.25 to 1 μg/ml; ciprofloxacin, 0.03 to 0.06 μg/ml; tetracycline, 4 to 8 μg/ml; meropenem, 0.03 to 0.06 μg/ml; azithromycin, 8 to 16 μg/ml; nalidixic acid, 8 to 16 μg/ml; cefotaxime, <0.25 μg/ml; chloramphenicol, <8 μg/ml; tigecycline, 1 to 2 μg/ml; ceftazidime, <0.05 μg/ml; colistin, 1 to 2 μg/ml; ampicillin, <1 to 2 μg/ml; gentamicin, 1 to 2 μg/ml. Strain 197 showed resistance to sulfamethoxazole (>512 μg/ml), trimethoprim (>32 μg/ml), tetracycline (>32 μg/ml), nalidixic acid (64 μg/ml), and ampicillin (>32 μg/ml).

### General genomic features and *in silico* serotyping.

WGS of 74 *S.* Dublin strains resulted in an average of 1,804,798 reads per sample (range, 912,103 to 2,912,074 reads per sample), with an average read length of 230 bp (see Data Set S1), leading to an average read coverage of 84-fold (range, 37- to 139-fold). The assembled genomes consisted of 29 contigs, on average (range, 24 to 115 contigs). The genome size of the strains was 4,878,835 bp, on average (range, 4,833,405 to 5,034,978 bp).

WGS-based serotyping performed by SISTR and SeqSero correlated with traditional serotyping predicting the formula 9:g,p:− for serovar *S*. Dublin for all strains (see Data Set S1). Classic multilocus sequence typing (MLST) revealed sequence types (STs) ST10 and ST3734, which is a single-locus variant of ST10 in the gene *sucA* (strain 671). The majority of the strains (69/74 strains) were assigned to ST10, and only one sample (strain 713) was assigned to ST3734. In the case of four strains (strains 523, 921, 1720, and 2218), a ST could not be assigned.

### AMR genes.

All *S*. Dublin strains harbored the chromosomal gene *aac(6′)-Iaa* for aminoglycoside resistance. Only strain 197 from the data set presented a multidrug resistance (MDR) pattern according to ResFinder, NCBI, and AMRFinder databases (see Data Set S2). The MDR pattern consisted of six resistance genes, i.e., *aac(6′)-Iaa* and *aph(6)-Id* for aminoglycoside resistance (streptomycin), *dfrA14* for trimethoprim resistance, *sul2* for sulfonamide resistance, *tet*(A) for tetracycline resistance, and *bla*_TEM-1_ for β-lactamase resistance. No chromosomal point mutations connected to AMR were found among the strains except for the isolates 89 and 1065, which were positive for a point mutation in the gene *acrB* (*acrB*-R717Q) that might cause macrolide (azithromycin) resistance according to AMRFinder.

### Virulence determinants.

Of the 22 Salmonella pathogenicity islands (SPIs) searched, 13 were identified within all 74 *S*. Dublin strains, including SPIs 1 to 6, 9, 11, 13, 14, 16, 17, and 19 (see Data Set S2). Regarding virulence determinants associated with the *S.* Dublin invasome, all strains presented the *spv* operon (*spvRABCD* genes), as well as the putative virulence gene *st313-td* (GenBank accession no. NC_016854), the type VI secretion system (T6SS) (accession no. NC_011205.1), and the lamboid prophage Gifsy-2 (GenBank accession no. NC_010393.1) (56% coverage). Among the other virulence determinants detected, the majority of strains (63/74 strains) revealed a total of 109 virulence genes, 10/74 isolates harbored less than 109 genes, and 1 strain (strain 2303) presented a virulence pattern of 114 genes (see Data Set S2). The 80-kb serovar-specific virulence plasmid pSDV (GenBank accession no. DQ115388.2) was found in all 74 isolates (see Data Set S2).

### Phylogenetic analysis and clustering.

**FIG 1:**
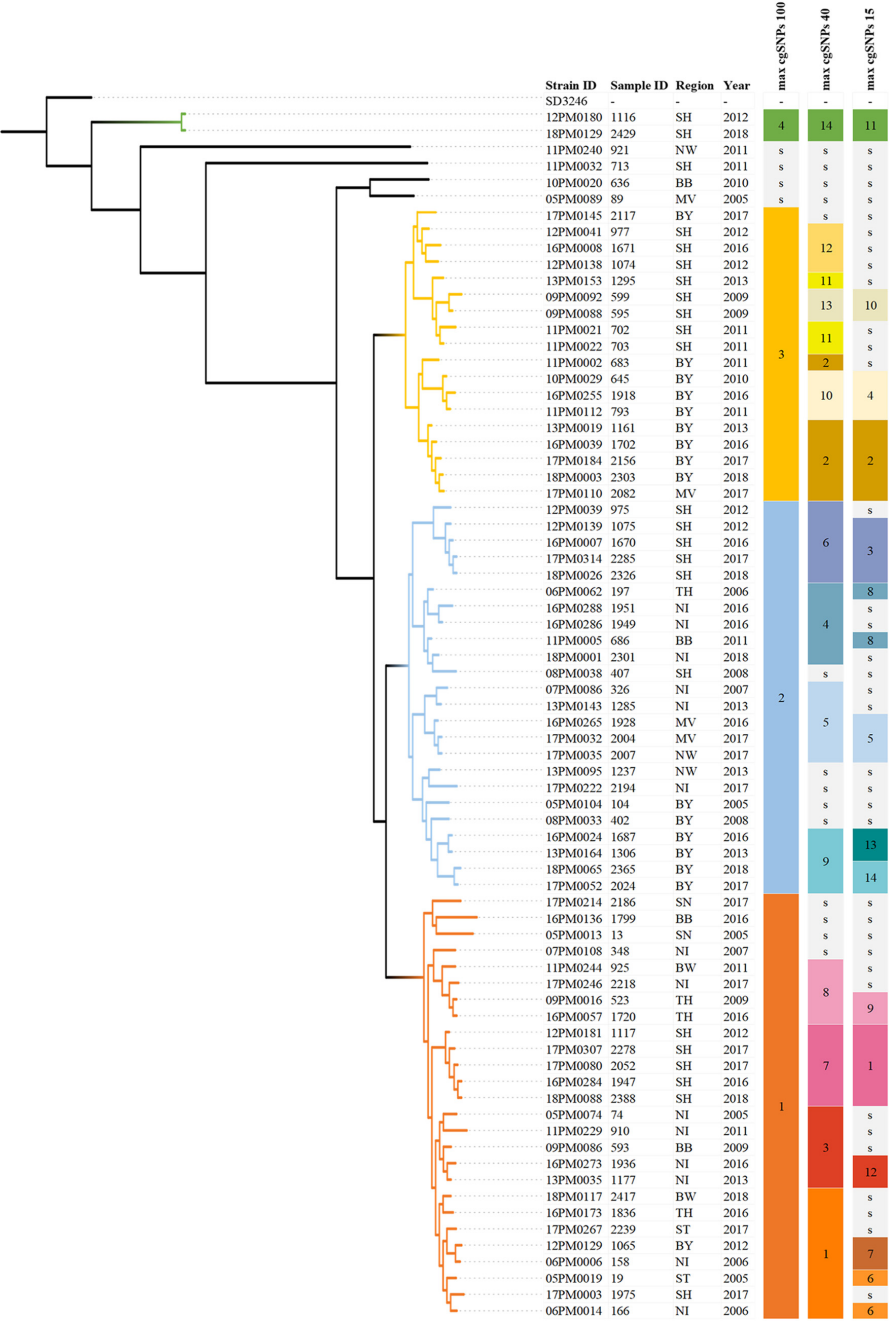
Maximum-likelihood phylogenetic tree based on cgSNP distances for 74 German Salmonella enterica subsp. *enterica* serovar Dublin strains. At the right, colored scales show the hierarchical clustering levels using cutoff values of 100 SNPs, 40 SNPs, and 15 SNPs. BB, Brandenburg; BW, Baden-Württemberg; BY, Bavaria; MV, Mecklenburg-Western Pomerania; NI, Lower Saxony; NW, North Rhine-Westphalia; SH, Schleswig-Holstein; SN, Saxony; ST, Saxony-Anhalt; TH, Thuringia.

To establish in-depth phylogeography of *S.* Dublin in Germany, cgSNP calling and cgMLST were performed. The cgSNP alignment consisted of 2,068 bp. The pairwise average cgSNP distance between German strains was 119 SNPs (range, 1 to 592 SNPs). Using cgMLST, the genome sequences of the German *S.* Dublin strains were mapped onto 3,002 predefined cgMLST target loci. For all 74 strains examined, an average of 98.4% of the targets were classified as “good targets” by Ridom SeqSphere (range, 97.4% to 98.6%). The average pairwise distance between strains was 68 alleles (range, 0 to 311 alleles) (see Data Set S3). For a higher resolution of *S*. Dublin phylogeny, we constructed a maximum-likelihood phylogenetic tree based on cgSNPs (Fig. 1) and a minimum spanning tree (MST) based on cgMLST. Both methods indicated (i) the occurrence of highly similar strains in very different regions in Germany and (ii) specific clusters of *S.* Dublin strains in only certain regions.

To study the phylogeography of *S*. Dublin in Germany in more detail, clustering of cgSNP and cgMLST results was performed to identify general genomic clusters (100 cgSNPs), intermediate clusters (40 cgSNPs), and more closely related strains (15 cgSNPs) (Fig. 1). For clustering based on cgMLST results using the MST, the same cutoff values as for cgSNP clustering were used (see Data Set S4). In general, the two methods provided similar results. Clustering based on 15 allelic differences in cgMLST, compared with clustering based on 15 cgSNPs, revealed mainly concordance; in only a few cases, cgMLST-based clusters were larger (see Data Set S4).

**FIG 2:**
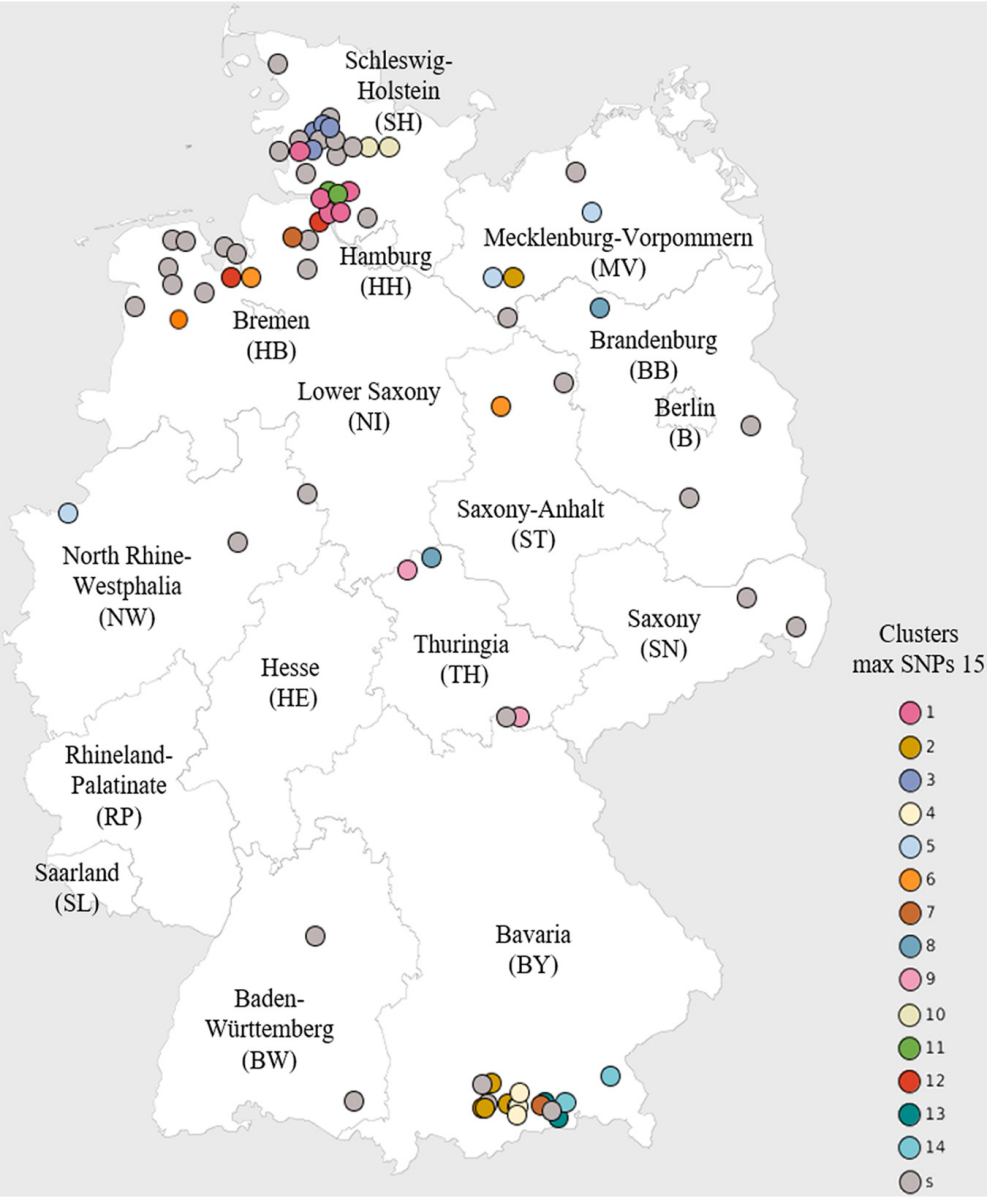
Geographical map of the German federal states, showing the origin of the 74 Salmonella enterica subsp. *enterica* serovar Dublin strains used in this study. Each color represents a different cluster according to the hierarchical cgSNP-based clustering with a cutoff value of 15 SNPs.

Both core-genome methods revealed higher resolution than classic MLST, as indicated by a larger Simpson’s index of diversity, which increases with a higher resolution of clustering (see Data Set S5). With a cutoff value of 100 cgSNPs, 70/74 strains clustered into four major clades (clades 1 to 4), and 4 strains were considered outliers (Fig. 1). These four outliers were at least 103 cgSNPs distant from any other sample. Cluster 1, based on 100 cgSNPs, contained strains that were isolated between 2005 and 2018. These organisms originated from all federal states considered in this study except for Mecklenburg-Western Pomerania and North Rhine-Westphalia (Fig. 1). Cluster 2 contained 24 samples that also were isolated between 2005 and 2018; they were isolated in Bavaria (6), Schleswig-Holstein (6), Lower Saxony (6), North Rhine-Westphalia (3), Mecklenburg-Western Pomerania (2), and Brandenburg (1) but not in Saxony, Saxony-Anhalt, or Baden-Württemberg. Cluster 3 contained 18 *S*. Dublin strains that were isolated between 2009 and 2018 only in Bavaria (9), Schleswig-Holstein (8), and Mecklenburg-Western Pomerania (1). Cluster 4 contained only 2 identical *S*. Dublin strains, which were isolated in Schleswig-Holstein in 2012 and 2018. Those 2 samples are most distant from any other sample (Fig. 1 and 2). In fact, they are separated from the nearest sample by 254 cgSNPs.

With a maximum distance of 40 cgSNPs, *S*. Dublin strains were grouped into 14 clusters and 14 singletons. Some clusters could be allocated to single federal states (clusters 6, 7, 11, 12, and 13 only in Schleswig-Holstein and clusters 9 and 10 in Bavaria) or were detectable in numerous federal states (clusters 1, 3, 4, 5, and 8) without a more frequent occurrence in certain regions. Except for strain 2082 from Mecklenburg-Western Pomerania, cluster 2 was found only in Bavaria. The 14 singleton strains within the clustering based on 40 cgSNPs originated from very different regions, all together from 7 federal states (Fig. 1).

Using a maximum distance of 15 cgSNPs, clustering of *S*. Dublin strains should give detailed information on their epidemiological links or contacts. Using 15 cgSNPs as the cutoff value revealed 14 clusters and 36 strains that were considered singletons. Nine clusters (clusters 1, 3, 4, and 9–14) grouped highly similar isolates collected from the same federal state (Fig. 1 and 2). Cluster 1, for example, was detected only in Schleswig-Holstein, over the period from 2012 to 2018. Strains from cluster 4 were found in Bavaria only but also over a period of 6 years. Five clusters (clusters 2 and 5–8) also comprised highly similar isolates; however, strains in these clusters were detected in different federal states. *S.* Dublin strains considered as singletons (36/74 strains) using 15 cgSNPs as the cutoff value occurred both in regions with higher numbers of *S*. Dublin outbreaks (Schleswig-Holstein, Lower Saxony, and Bavaria) and in regions with rarely occurring outbreaks (North Rhine-Westphalia, Saxony-Anhalt, Brandenburg, Thuringia, Saxony, and Baden-Württemberg) (Fig. 1 and 2). Moreover, using a maximum distance of 0 cgSNPs (see Data Set S4), two clusters with two strains each were identified, which were isolated either in different years in Schleswig-Holstein (strains 1947 and 2052) or in different years and different federal states (Mecklenburg-Western Pomerania and North Rhine-Westphalia) (strains 2004 and 2007).

### Comparison of *S.* Dublin genomes from Germany, Sweden, and Denmark.

The genomes of the 74 German *S.* Dublin strains were compared with raw sequence data from 197 *S*. Dublin isolates from Denmark (13) and 28 strains from Sweden (11). Classic MLST detected ST10 for all isolates from Denmark and Sweden except for 2 isolates from Sweden with unknown ST (see Data Set S6). ST10 was also the most abundant ST (69/74 strains) in the German data set. The cgSNP alignment between the German data set and the data from Denmark and Sweden (*n* = 299) had a length of 1,680 bp and a pairwise average core SNP distance of 107 cgSNPs (range, 0 to 284 cgSNPs), excluding the reference strain. Regarding allele distance, cgMLST analysis revealed an average of 154 alleles (range, 0 to 384 alleles), without considering the reference strain (see Data Set S3). To study the relationship between the German data set and public genomes from Denmark and Sweden, a maximum-likelihood tree and an MST were constructed. Both methods revealed three main clusters, i.e., clusters A, B, and C. Cluster A contained 101 isolates representing the majority (92%) of the German strains but also 6 strains from Denmark and 28 strains from Sweden. The German-derived strains formed their own subcluster within cluster A, in which only 2 isolates (strains 89 and 636) were nearest neighbors to the Danish population and 1 isolate (strain 713) to the Swedish population. In fact, the minimum distance from a German isolate to an isolate not from Germany inside cluster A was 58 cgSNPS when considering strain 89 and strain ERR3491245 from Denmark. Cluster B grouped 42 Danish strains and 2 German isolates (strains 1116 and 2429), which were 58 cgSNPs or 59 cgSNPs away from the closest Danish strains (the clonally related strains ERR3491299 and ERR3491381), respectively. Clade C comprised 76% of the Danish isolates (149/197 strains) and 1 German strain (strain 921) at a distance of at least 141 cgSNPs.

## DISCUSSION

In the present study, WGS was used to decipher the genetic traits and phylogeny of the bovine host-adapted serovar *S*. Dublin in Germany. Although the majority of Salmonella serovars can be correctly predicted using bioinformatic tools based on WGS data, a certain number of serovars cannot be determined exactly (16, 17). For *S*. Dublin, genoserotyping seems to have no limitations; for all strains analyzed, genoserotyping correlated completely with conventional serotyping. In addition, this study reveals a close agreement between the genotypic prediction of resistance genes and phenotypic antimicrobial susceptibility. Although several clones of different Salmonella serovars resistant to fluoroquinolones and third-generation cephalosporins are globally emerging (18), resistance to those compounds has not yet been detected in *S*. Dublin isolates from cattle and calves (19). In this study, 73 of 74 *S.* Dublin strains were not resistant to the antimicrobial substances tested. An MDR pattern consisting of resistance to aminoglycosides, trimethoprim, sulfonamides, tetracyclines, and β-lactamases was detected in only 1 of the *S.* Dublin strains included. Therefore, the occurrence of MDR *S.* Dublin organisms in the German cattle population is uncommon, confirming earlier findings (5, 6) and results from other countries in Europe (13). In contrast, in the United States, *S.* Dublin has become one of the most important MDR serovars (20, 21).

WGS has been applied to identify the virulence factors that integrate into the *S*. Dublin invasome and that play a role in the pathogenicity of this serovar (22). The *spv* (Salmonella plasmid virulence) locus in *S.* Dublin is of special interest because it contributes to enteritis as well as lethal disease in young calves (21, 23). It has been identified in the approximately 80-kb serovar-specific virulence plasmid commonly found in this serovar (18, 24–26). Both the *spv* operon and the serovar-specific virulence plasmid *spv* were detected within the 74 German *S*. Dublin genomes. SPIs are large genetic elements harboring important virulence factors. For example, SPI-1 plays a crucial role in the invasion of host cells and interactions with the host immune system (27). In this study, 13 SPIs were detected within all 74 strains; among them, SPI-1 but also SPI-6 and SPI-9 received special attention, because they encode the T6SS that forms part of the *S*. Dublin invasome (22). The occurrence of the high number of SPIs in all strains analyzed indicates rather low diversity between strains. This is supported by limited sequence variation between *S.* Dublin genomes due to the slow single-gene gain and loss and the low rate of cgSNPs differences (13); thus, reduced diversity of *S.* Dublin genomes over time is suggested (11).

Because the phylogeography of *S*. Dublin in Denmark and Sweden has recently been analyzed using WGS, these data were used to compare the German results with those for neighboring European countries (11, 13, 28). Although there is country-specific phylogeny, the numbers of detected cgSNPs are comparable. Similar to Germany, there are regionally specific clones on one hand and clones spreading all over the country on the other hand (13). Within the analyzed data set, there is no close similarity between German strains and Danish or Swedish isolates. Therefore, exchange of *S*. Dublin strains between Germany and the other two countries has not happened or has happened only occasionally.

In this study, cgSNP typing and cgMLST were used to understand the epidemiological context of *S.* Dublin infections in German cattle herds. Information on genetic characteristics from WGS should be utilized to analyze routes of spreading, transmission, or persistence of *S.* Dublin within and between regions or federal states in Germany over time. However, the use of core genome sequence information for this purpose leads to a number of challenges, especially identification of the required level of relatedness between isolates to characterize a clone (29). WGS-based studies on *S.* Dublin used different cgSNP thresholds to identify closely related strains (11, 13, 28). However, one of the aims of this study was not only identification of clonal organisms within or between single outbreaks but also evaluation of the epidemiological *S*. Dublin situation in Germany over a prolonged period. The special characteristics of *S*. Dublin, the high degree of host adaptation, and the rather low rate at which the serovar mutates at the nucleotide level (30) enable the identification of these epidemiological aspects. Compared to non-host-adapted Salmonella serovars occurring in cattle, spreading of bovine host-adapted *S.* Dublin between cattle farms is nearly unanimously associated with the transport or movement of infected animals (30). In the case of other serovars, the first entry of salmonellae into farms may be caused by a large number of biotic and abiotic factors, and thus epidemiological studies on the spreading of those serovars may be much more difficult. Therefore, not only thresholds of 15 cgSNPs but also cutoff values of 40 cgSNPs and 100 cgSNPS were considered to analyze the cattle-derived *S.* Dublin organisms from different regions and different points of time.

In fact, even using 100 cgSNPs two different clusters with strains isolated over the whole study period from 2005 to 2018 from nearly all regions with outbreaks of *S.* Dublin were identified. However, a third cluster contained only strains from two federal states. These findings indicate (i) a rather nationwide occurrence of two general clusters over longer periods and (ii) also a limited spread or persistence of a third cluster with possible exchange of strains between regions in and between federal states. In the case of 40 cgSNPs, clusters became evident showing both (i) the occurrence in one federal state over various periods, indicating a persistence of the infection over time by more related organisms, and (ii) the occurrence in different federal states, indicating both persistence and probable exchange of the organisms between the federal states. Most interesting in the case of 40 cgSNPs is cluster 1, which includes 8 strains from six federal states; therefore, the question arises of whether any conclusion on spreading of the strains from this cluster is justified. A maximal distance of 15 cgSNPS between *S.* Dublin strains is considered to identify epidemiological links between cattle herds (13, 30). Using this value, several epidemiological aspects not only can be suspected but in fact can be strongly assumed. This refers to the detection of outbreaks in one region in rather short periods (1 year), as well as in very long periods (up to 5 years [e.g., in clusters 2 and 3]). Furthermore, the occurrence of highly related *S.* Dublin strains in very different and distant federal states at different times indicates a possible exchange of the organisms through the movement of animals. These indications by the cgSNP analysis give very valuable data; however, information on the management of the cattle farms and the contacts with associated farms or herds is essential to detect possible routes of infection or persistence of the organism. A number of strains are singletons in the 15-cgSNP clustering, indicating a missing close relationship of these strains to any other isolate in the data set examined. Detailed conclusions regarding routes of transmission, persistence, or contacts between herds for these strains cannot be drawn; however, the information based on 40-cgSNP or 100-cgSNP clustering provides findings on their phylogeography, such as their occurrence in regional clusters.

Moreover, the use of different thresholds for cgSNP analysis in this study enabled a very broad and also detailed view of the occurrence of *S.* Dublin in Germany. The persistence of a rather large cluster in exclusively the same regions in the federal states Schleswig-Holstein and Bavaria over very long time periods clearly indicates a strong persistence and a permanent exchange of the closely related *S.* Dublin organisms within these rather restricted regions. Therefore, control strategies in place seem not to be effective enough to interrupt the existing routes of infection. Apart from the movement of infected animals, attention to prevent the spread of *S.* Dublin in special regions must also be given in the use of common vehicles, the exchange of manure, or the use of shared pastures (31). As also shown in this study, a transfer of persisting *S.* Dublin strains in other, distant regions may occur but obviously not frequently. Final evidence on these transmission routes might be gained by the analysis of both cgSNPs at a cutoff value of ≤15 and data on farming management of the affected cattle herds. Therefore, WGS proved to be an excellent tool for epidemiological studies; however, complex epidemiological analyses require additional information on cattle farming practices. Nonetheless, results from this study also confirm that restriction of movement of *S.* Dublin-infected cattle represents the most effective strategy to prevent regional and nationwide spread of *S.* Dublin.

## MATERIALS AND METHODS

### Bacterial strains.

In this study, we analyzed a data set composed of 74 *S.* Dublin strains covering all federal states in Germany in which this serovar has been detected, over the period between 2005 and 2018. Each strain originated from a single farm with a proven outbreak of salmonellosis in cattle officially confirmed by a competent authority. Because *S.* Dublin is predominantly provable in only a few federal states and not consistently in all German regions, strains from those federal states (e.g., Bavaria, Schleswig-Holstein, and Lower Saxony) contributed in greater numbers to the data set analyzed. Other states (e.g., Thuringia, North Rhine-Westphalia, and Saxony) contributed only a few isolates (Fig. 1 and 2). The annual variation in the number of *S.* Dublin outbreaks in the different regions has also been considered in the selection of the strains examined. The selected *S.* Dublin isolates originated from the collection of bovine host-derived Salmonella strains at the NRL for Salmonellosis in Cattle in Germany (see Data Set S7 in the supplemental material). The NRL receives Salmonella organisms isolated at cattle farms in Germany with suspected or confirmed outbreaks of salmonellosis from the investigation offices in the federal states for further characterization.

### Serotyping and antimicrobial susceptibility testing.

All Salmonella strains were serotyped using polyvalent and monovalent anti-O and anti-H sera (SIFIN, Germany) according to the Kauffmann-White scheme (32). Antimicrobial susceptibility of the *S*. Dublin strains was assessed by determining the MICs using the broth microdilution method with Sensititre EUVSEC plates (Trek Diagnostic Systems Ltd., East Grinstead, UK). Epidemiological cutoff values according to the European Committee on Antimicrobial Susceptibility Testing (EUCAST) (33) were used. Antimicrobial susceptibilities to sulfamethoxazole, trimethoprim, ciprofloxacin, tetracycline, meropenem, azithromycin, nalidixic acid, cefotaxime, chloramphenicol, tigecycline, ceftazidime, colistin, ampicillin, and gentamicin were examined.

### WGS.

Genomic DNA of 74 *S*. Dublin strains was prepared using the Qiagen Genomic-tip 20/G kit (Qiagen, Germany) and the genomic DNA buffer set (Qiagen, Germany). The concentration of the DNA was determined using the Qubit double-stranded DNA (dsDNA) broad-range (BR) assay kit (Invitrogen, USA). Sequencing libraries were prepared using the Nextera XT DNA library preparation kit (Illumina, Inc., USA). Paired-end sequencing of the *S.* Dublin strains was performed with a MiSeq instrument (Illumina) according to the manufacturer’s instructions.

### Analysis of *S*. Dublin genomes from Sweden and Denmark.

To put the findings into context with Germany’s neighboring countries, recent studies performed in Denmark (13, 30) and Sweden (11) were selected for analysis. Paired-end Illumina sequencing data (last accessed June 2020) from Sweden (*n* = 28) (see Data Set S7) and Denmark (*n* = 197) (see Data Set S7) were downloaded from the European Nucleotide Archive and the NCBI Sequence Read Archive (SRA).

### Data quality control and *in silico* serotyping in bioinformatic analyses.

Raw paired-end reads from Illumina sequencing were used as input for the Linux-based bioinformatics pipeline WGSBAC v. 2.0.0 (34) to perform the data analysis, as recently published (14). First, WGSBAC uses FastQC v. 0.11.7 (35) to perform read quality control. The raw coverage, defined as the number of reads by their average read length divided by the genome size of the reference genome, is estimated. WGSBAC performs assembly using Shovill v. 1.0.4 (36) implementation of the SPAdes assembler (37). To check the quality of the assembled genomes, WGSBAC uses QUAST v. 5.0.2 (38). To classify sequences, and thus to identify potential contamination in both reads and assemblies, the pipeline uses Kraken 2 v. 1.1 (39) and the database Kraken2DB. For *in silico* serotyping, WGSBAC relies on SISTR v. 1.0.2 (16) for serovar prediction using the assembled genomes and SeqSero, which works using genome assemblies as well as raw sequence reads (40).

### *In silico* AMR genes and virulence determinants.

For detection of AMR genes and virulence determinants, WGSBAC uses the software ABRicate v. 0.8.10 (41) and the databases ResFinder (42), NCBI (43), Virulence Factor Database (VFDB) (44), and PlasmidFinder (45). In addition, WGSBAC uses AMRFinderPlus v. 3.6.10 (46) for the detection of chromosomal point mutations leading to AMR and organism-specific acquired resistance genes. For the identification of SPIs, sequences for 22 SPIs were downloaded from the public data repositories Pathogenicity Island Database (PAIDB) (47) and NCBI (48) and used to create a customized database for ABRicate (https://gitlab.com/FLI_Bioinfo_pub/spis_ibiz_database). A cutoff value of 60% coverage was applied to consider a strain positive for a SPI. In addition to VFDB, customized ABRicate databases were used to detect *S*. Dublin-specific virulence determinants that are part of the invasome, i.e., (i) the *spv* (Salmonella plasmid virulence) operon (including *spvRABCD* genes), (ii) the gene *st313-td* for flagellum biosynthesis, and (iii) putative virulence determinants such as the T6SS, the lambdoid Gifsy-2 prophage, and the *fae* cluster (coding for fimbria biosynthesis) (22). BLAST was used to detect the serovar-specific virulence plasmid pSDV (GenBank accession no. DQ115388.2).

### Genotyping, phylogenetic analysis, and clustering.

For genotyping, WGSBAC uses classic MLST of assembled genomes using the software mlst v. 2.16.1 (49), which incorporates the PubMLST database for the 7-gene Salmonella MLST scheme (https://pubmlst.org/salmonella) (50). cgMLST was performed using the external software Ridom SeqSphere+ v. 5.1.0 (51) with default settings, including definition of good cgMLST targets as follows: (i) same length as reference genes plus or minus three triplets, (ii) no ambiguities, and (iii) no frameshifts. The specific core genome scheme (cgMLST v. 2) for Salmonella enterica with 3,002 target loci developed by EnteroBase (52) was used within Ridom SeqSphere. To infer phylogeny based on cgMLST results, an MST and a geographical map were depicted and visualized using the external software Ridom SeqSphere+ v. 7.1.0 (https://www.ridom.de/seqsphere). Furthermore, WGSBAC performs mapping-based genotyping using cgSNPs identified by Snippy v. 4.3.6 (53) with standard settings and as reference the complete genome of *S.* Dublin strain 3246 (GenBank accession no. CM001151). For phylogenetic tree construction based on cgSNP analysis, WGSBAC uses the SNP alignment matrix generated by Snippy and reconstructs the tree using RAxML v. 8 (54). The tree was rooted to the reference genome and visualized using the interactive Tree of Life (iTOL) v. 4 web tool (55) (https://itol.embl.de). The pairwise SNP distance between isolates was calculated using the tool snps-dists v 0.63. For hierarchical clustering based on the cgSNP alignment, WGSBAC computes classic hierarchical clustering based on the SNP distance matrix from Snippy using the hierClust function v. 5.1 of R. For clustering based on cgMLST results, cluster calculation based on allele distance was performed by Ridom SeqSphere+, and the same cutoff values as for cgSNPs were applied. As previously applied (13), a cutoff value of 15 cgSNPs was applied to identify closely related strains. In addition, a cutoff value of 100 cgSNPs was chosen to identify more general clusters and 40 cgSNPs for intermediate clusters. Simpson´s index was calculated using the web-based tool Comparing Partitions (http://www.comparingpartitions.info/index.php?link=Home).

### Data availability.

Raw sequencing data were deposited in the NCBI SRA under the BioProject accession number PRJNA678856.
